# COVID-19 Associated With Life-Threatening Apnea in an Infant Born Preterm: A Case Report

**DOI:** 10.3389/fped.2020.00568

**Published:** 2020-09-15

**Authors:** Gauthier Loron, Thibault Tromeur, Perrine Venot, Jonathan Beck, Laurent Andreoletti, Pierre Mauran, Nathalie Bednarek

**Affiliations:** ^1^Department of Pediatrics, CHU Reims, Reims, France; ^2^CReSTIC / EA 3804, Reims Champagne-Ardenne University, Reims, France; ^3^Medical University Reims Champagne-Ardenne University, Reims, France; ^4^Cardiovir, EA 4684, Reims Champagne-Ardenne University, Reims, France; ^5^Department of Virology, CHU Reims, Reims, France; ^6^Pediatric Cardiology Unit, CHU Reims, Reims, France

**Keywords:** SARS-CoV-2, coronavirus, apnea, case report, children

## Abstract

A pandemic linked to the new coronavirus strain (SARS-CoV-2) has been raging for several months. Pediatric populations are less impacted than adults, and critical respiratory diseases seem rare (1, 2). We report the case of an infant, who presented with life-threatening apneas at home requiring basic life support. SARS-CoV-2 was subsequently identified in the patient's nasopharyngeal aspirate. He did not present with bronchiolitis or hypoxic failure as described in severe forms of COVID−19. The outcome was favorable in a few hours. The occurrence of apneas is not uncommon during viral respiratory infections in early infancy; however, there are very few descriptions related to a documented SARS-CoV-2 respiratory tract infection. In light of this clinical case, it seems necessary to quickly bring up a potential COVID-19 contamination in infants admitted for life-threatening apnea, in order to properly report and isolate these patients to avoid further nosocomial dissemination of SARS-CoV-2.

## Background

In December 2019, the Chinese authorities notified the World Health Organization of a pneumonia outbreak of unknown etiology in the city of Wuhan (Hubei area). A novel Coronavirus, designated as SARS-CoV-2, was promptly characterized and identified as the causative agent of this new respiratory illness, identified as COVID-19.

Within 6 months, more than 11.10^6^ confirmed cases and 500,000 death were reported worldwide (3).

At the beginning of July 2020, confirmed COVID-19 cases and related deaths exceeded 78,000/4,600, 166,000/29,000, and 28,400,000/129,500 cases in China, France, and USA, respectively (4–6).

Clinical features of COVID-19 are diverse and range from asymptomatic to mild respiratory tract disease, severe progressive pneumonia, acute hypoxia, severe respiratory distress syndrome (ARDS), and death (7).

As described in other coronavirus epidemics, children seem relatively less affected than adults (8, 9).

Among pediatric cases, asymptomatic forms are more frequent, and critical respiratory cases remain rare (1, 2, 10–12).

However, in children under 1 year of age the proportion of severe forms is higher than older children, the mortality rate is also slightly higher (1, 8, 12).

In USA, the percentage of confirmed cases / mortality is 1.4% / 1.4% and 1.4 / 0.17% among children 0–4 and 5–17 years, respectively (13). Similarly, <1 percent of confirmed cases were reported in children below 10 years of age (4).

Herein, we report the case of an infant born preterm in whom the SARS-CoV-2 infection was revealed by life-threatening, recurrent apneas.

## Case Report

### Past Medical History: Neonatal Course

The mother of this boy, a 33-years-old woman, was admitted in our tertiary level center for severe preeclampsia at 29+4 gestational age (GA). Her significant medical history included morbid obesity, gravidity 7, parity 5. She received steroids and no pathogen was identified from the vaginal swab. A preterm boy weighing 1.400 g was extracted at 30+2 GA by emergency cesarean section due to the mother's worsening condition. Resuscitation required manual ventilation for 1 min, Apgar scores were 3, 8, 9 at 1, 5, and 10 min, respectively. CRIB-II score was quoted at six (Clinical Risk Index for Baby score, increasing severity from 0 to 27).

He subsequently presented with a mild hyaline membrane disease requiring nasal CPAP for 72 h then high-flow nasal canula for 8 days. He received Caffeine until 34+5 GA and was formula-fed.

He was discharged at home at 35+5 GA (mid-February), with a weight of 2.215 kg.

### History of the Disease

On March 5th, 2020, the mother experienced cough, anosmia, and chest pain. She stayed home and was not tested for SARS-Cov-2.

**Figure 1:**
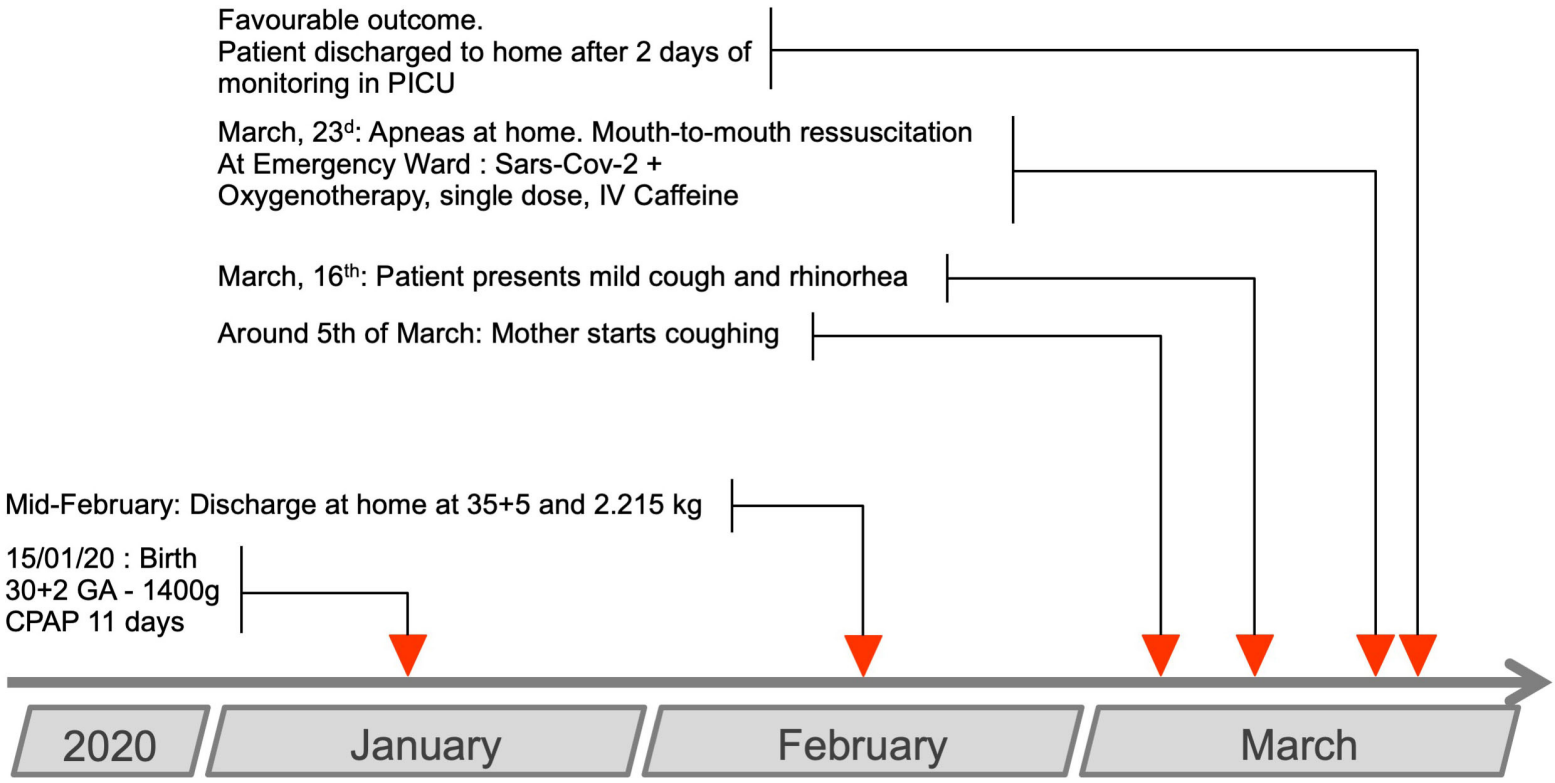
Case timeline.

Seven days later, the baby presented with mild cough, rhinorrhea, and abnormal paleness (Figure 1). On March 23, 2020, the parents noticed unusual fatigue. In the morning he presented with non-bilious, food vomiting. After the bath, away from a meal, while his mother carried him on her chest, his head on her shoulder, she noticed a change in the baby's behavior and absence of reaction. Looking at him she discovered extreme cyanosis. No respiratory movement was discernable. She initiated mouth-to-mouth resuscitation and chest compressions while the father called for a rescue unit. A neighbor came to help the mother. The baby recovered a spontaneous, yet irregular ventilation after around 3 min of basic life support. At the arrival of the emergency rescue team, the airway was free from obstruction or vomit stain. The baby had recovered spontaneous breathing, albeit recurrent apneas were observed, requiring tactile stimulation. The SpO_2_ level was at 70%. Heart rate was 175 bpm, blood pressure at 107/92 mmHg. He was quickly transferred to the emergency department of our tertiary level center, under nasal oxygen supply (1 L/min, SpO_2_ 100%).

### Diagnostic Assessment

Upon admission to the emergency ward, the heart rate was 140 bpm, blood pressure at 81/49 mmHg, temperature 37.3°C, capillary refill time 2 s, pulse oxygen saturation 100% under oxygen (nasal canula, 1 L/min). Respiratory rate was 52 breaths per minute except for unexpected, recurrent apneas responding to stimulation. Mild cough and non-obstructive rhinitis were reported, without grunting or the involvement of accessory muscles.

The cardiac and pulmonary auscultation was normal, without any wheezing or rales.

Arterial blood tests showed: pH 7.3, PaCO_2_ 63 mmHg, HCO_3_ 32 mmol/L, lactic acid 2.4 mmol/L (*N* < 2,5 mmol/L), CPK 373 UI/L (*N* < 308), ASAT 206 UI/L (*N* < 50), ALAT 120 UI/L (*N* < 50), GGT 135 UI/L (*N* < 61), Na 146 mmol/l, K 4.6 mmol/L, Ammonia 53 μmol/L, glycemia 2.4 mmol/L, CRP 1.3 mg/L, PCT 0.12 ng/ml, Hb 82 g/L, Leucocyte 13.3 G/L (lymphocyte 8.1 G/L), platelets 516 G/L. Urine analysis showed no infection.

Hypercapnia and elevated bicarbonates suggested that the subacute ventilatory impairment might have occurred some hours/days before this life-threatening apnea. However, after a careful questioning of the parents no respiratory distress or nasal obstruction was noted in the days preceding the apneas, there was however a mild rhinitis. The baby had not exhibited gastroesophageal reflux disease so far.

Electrocardiogram, cardiac and cerebral ultrasound examinations were normal, excluding congenital or acquired cardiac disease and brain disorders (seizures, subdural bleeding). Chest X-rays were normal with no sign of lung disease, aspiration syndrome, atelectasis or ground-glass opacification.

The nasopharyngeal aspirate sample was negative using a rapid multiplex RT-PCR assay for the detection or Respiratory Syncytial Virus, Influenza viruses A and B (Xpert FLU/RSV XC kit, Cepheid®) and for the molecular detection of Bordetella Pertussis (RealCycler BordGX kit, Progenic Molecular®).

SARS-CoV-2 RNA was detected in the same sample using a referenced RT-PCR assay allowing us to validate the diagnosis of COVID-19 infection (14).

A single 20 mg/kg dose of intravenous caffeine was administered.

The patient was transferred to the pediatric intensive care unit for close monitoring. No apnea was observed after the single caffeine dose. Oxygen therapy was progressively weaned over a 12-h period.

The baby was discharged home 48 h later.

Five days later, the father was hospitalized with a documented, severe SARS-CoV-2 infection, that required hospitalization and non-invasive ventilation.

The neighbor, who had come to help during the initial episode, developed documented, mild SARS-CoV-2 infection in the following days.

Six weeks later, as the first myocarditis and Kawasaki-like symptoms were reported and suspected to be consecutive to COVID-19, the baby benefited from a cardiac ultrasound. This was normal.

## Discussion

We reported the clinical features of a child, born prematurely, who developed life-threatening apnea in association with a COVID-19 infection.

Regarding the central or peripheral origin of the apneas in this report, no polysomnographic recording was done to validate their central origin. The rapid improvement of the patient and the availability of the electroencephalographic or polysomnographic technique during the pandemic period did not allow for this examination. However, several items point to a central origin:

- the absence of nasal obstruction: the rhinitis was mild and non-obstructive
- the finding of unexpected apneas without any respiratory effort observed by the emergency rescue team and emergency department caregivers,
- the plausibility: central apneas during a viral infection, especially in infants born prematurely, are not uncommon.

Indeed, as previously shown central apneas induced by viral infections can occur before the age of 2 months or before 48 weeks corrected age in former preterm infants (15). Isolated apneas, without cough/respiratory distress/fever were reported in 1.6–5% of respiratory infection cases. Syncytial respiratory virus, metapneumovirus, influenza, parainfluenza, as well as previously known coronavirus, were commonly involved (15, 16).

Regarding the causal link or simple association between life-threatening apneas and the identification of SARS-CoV-2, knowing the probability of false positives and the novelty of this observation, we put forward the following arguments:

- The common viruses causing respiratory infections in infants were not identified by RT-PCR.
- Several common differential diagnoses were ruled out by additional examinations (congenital or acquired heart disease, cerebral hemorrhage) and in light of the rapid improvement of the patient's clinical condition.
- A family cluster was documented.
- As seen previously, central apneas were already reported in other viral infections of the small child, including during coronavirus infections (16).

Several cases of mother-to-child transmission were described, although they remain rare (17, 18). The contamination of infants in family clusters was more often described, similarly to the case reported here (19). In addition, in this observation, bottle-feeding prevented the child from receiving maternal antibodies.

Regarding the putative pathophysiology of such apnea related to viral infections, it is established that cough is mainly driven by the laryngeal reflex arc. Experimentally, this reflex is assessed with the administration of a small bolus of fluid along the laryngeal wall. In young infants, such a stimulation results in apnea or larynx closure, rather than cough. The latter becoming the main reflex with maturation. During a viral infection of the upper airways, interleukins are secreted in the laryngeal mucosa, carried along axons by retrograde transport toward the brainstem respiratory centers. Those interleukins may sensitize the laryngeal reflex arc, inducing an “apnea-type” response in the most immature infants (20).

Caffeine is successfully used in the treatment of central apneas in premature infants. By extension, this treatment is sometimes used in central apneas due to early, respiratory infection, as in the present case. However, the level of evidence for caffeine efficacy is quite low, as respiratory support (high-flow nasal canula or jet-CPAP) is often conjointly administered (21).

To our best knowledge, very few articles have mentioned apneas associated with COVID-19 in children (22–24). The other symptoms presented by this child: dry cough, moderate rhinitis, fatigue, although non-specific, are commonly reported in the literature for COVID-19 infections (25). Some atypical convulsive seizures are reported (2), which may be related to the clinical presentation of this patient. The Chinese (1), Italian (2), and North American (10) series, as well as several meta-analyses and systematic reviews (11, 17) do not reference apneas in their reports.

When the first reports of pediatric inflammatory syndrome (PIMS) / multisystem inflammatory syndrome in children (MIS-C) were published, the patient promptly benefited from an echocardiography, which ruled out any residual myocardial dysfunction nor coronary dilatation. Indeed, the patient had not presented the commonly reported clinical features of PIMS/MIS-C (rash, features of myocardial dysfunction, shock, and elevated markers of inflammation) (26–29). Among the series published to date concerning PIMS/MIS-C, none mention the existence of apnea in the acute phase (26–30).

Our case shows that SARS-CoV-2 may cause apnea and brief resolved unexpected event (BRUE) in very young infants.

In the current pandemic context of this infection, a rapid identification of those patients is mandatory to implement appropriate droplet precautions and contact isolation procedures to avoid nosocomial infections in pediatric wards, or contamination of caregivers.

Once again, it is essential to remind families of appropriate measures to prevent respiratory infections in premature babies returning home.

Moreover, vigilance is required regarding the frequency of these serious symptoms in younger, formerly premature infants. These children and/or their parents, could be a target population for a vaccination strategy.

## Data Availability Statement

The original contributions presented in the study are included in the article, further inquiries can be directed to the corresponding author/s.

## Ethics Statement

Written informed consent was obtained from the minor(s)' legal guardian/next of kin for the publication of any potentially identifiable images or data included in this article.

## Author Contributions

GL have collected data, drafted, revised, and submitted the manuscript. TT and PV have collected data and drafted the article. JB, LA, and PM have contributed to manuscript revisions and edition. NB have designed the case report and supervised the revision process. All authors have given final approval of the version to be published and agreed to be accountable to all aspects of the work.

## Conflict of Interest

The authors declare that the research was conducted in the absence of any commercial or financial relationships that could be construed as a potential conflict of interest.

## Abbreviations

BRUE: Briefly Resolved Unexpected Event
GA: Gestational age
ARDS: Acute respiratory distress syndrome.
